# Involving Children With Cancer in Health Promotive Research: A Case Study Describing Why, What, and How

**DOI:** 10.2196/resprot.7094

**Published:** 2017-02-07

**Authors:** Jens M Nygren, Susanne Lindberg, Pontus Wärnestål, Petra Svedberg

**Affiliations:** ^1^ School of Health and Welfare Halmstad University Halmstad Sweden; ^2^ School of Information Technology Halmstad University Halmstad Sweden

**Keywords:** children, participation, involvement, research

## Abstract

**Background:**

Participatory research approaches have been introduced to meet end-users’ needs in the development of health promotion interventions among children. However, whereas children are increasingly involved as passive informants in particular parts of research, they are rarely involved as partners, equal to adult researchers, throughout the research process. This is especially prominent in the context of child health where the child is commonly considered to be vulnerable or when the research concerns sensitive situations. In these cases, researchers and gatekeepers to children’s involvement base their resistance to active involvement of children on potential adverse effects on the accuracy or quality of the research or on ethical or moral principles that participation might harm the child. Thus most research aimed at developing health promotion interventions for children in health care is primarily based on the involvement of parents, caregivers, and other stakeholders.

**Objective:**

The objective of this paper is to discuss reasons for involving children in health promotive research and to explore models for children’s participation in research as a basis for describing how researchers can use design methodology and participatory approaches to support the participation and contribution of children in a vulnerable context.

**Methods:**

We developed and applied a model for children's participation in research to the development of a digital peer support service for children cancer survivors. This guided the selection of appropriate research and design methodologies (such as interviews, focus groups, design sessions, and usability evaluation) for involving the children cancer survivors (8-12 years) in the design of a digital peer support service.

**Results:**

We present a model for what children’s participation in research means and describe how we practically implemented this model in a research project on children with cancer. This paper can inform researchers in their planning of strategies for children’s participation and ensure future development of health promotion interventions for children is based on their perspectives.

**Conclusions:**

Challenges in reaching a suitable degree of participation during a research project involve both creating opportunities for children to have genuine influence on the research process and organizing this involvement so that they feel they understand what they are involved in and why. To achieve this, it is essential to enable children to be involved in research over time to gain confidence in the researchers and to develop children’s abilities to make decisions throughout the research processes.

## Introduction

### Overview

In Sweden, approximately 300 children are diagnosed with cancer each year [1], but advances in diagnosis, risk stratification, and treatment protocols have resulted in most children surviving the disease. Increased survival rates have created new needs for treatment and support associated with physical and psychological late effects of the treatment—effects that may not manifest themselves until years later [2]. Managing these late effects as well as social challenges that are associated with the experiences and consequences of the disease can be facilitated by social support from peers who share similar experiences [3]. This notion is supported by observations that those who experience more social support within this target group report fewer symptoms of depression and anxiety [4]. The availability of peer support is, however, limited, and resources are often offered sporadically and at a limited number of geographically confined locations [5]. Digital peer support built on accessible and asynchronous technologies could potentially solve the feasibility limitations of face-to-face peer support. Designing such digital services is dependent on knowing the preferences and requirements of the target group [6]. Based on this, we set out to involve children cancer survivors in the design of a digital peer support service for children between the ages of 8 and 12 years who have been cured from cancer. Involving this user group in the design and evaluation of a digital peer support service is particularly challenging due to the built-in complexity of the context, coupled with vulnerability, gatekeeping, and availability. Children are often viewed as vulnerable due to their dependence on adults. It is therefore essential to protect children from harm associated with involvement in research and carefully handle consent to participation, confidentiality, research context, and activities [7]. The vulnerability of children is even more pronounced when it comes to children with illnesses, and it is common that their participation is restricted by various gatekeepers such as ethical boards, health care professionals, or parents [8]. The characteristics of the intended user group (eg, age span, medical history, geographic spread, and clinical restrictions) make it difficult to recruit, meet, and engage the children on a regular basis. Research processes and methods that take all these considerations into account could be powerful ways to involve children in the context of health promotive research such as that outlined above.

In recent years, there has been a growing body of academic articles describing involvement of children in research projects [8-11]. A few of these address methodological issues especially from the perspective of children with cancer. Different participatory research approaches have been introduced in order to meet children’s needs in the development of health promotive research. Even though all approaches have an end-user focus in common, they differ in regard to whether the children are passive partners in separate parts of the research project or if the children are active partners throughout the research process with real influence over the project. Thus, children are often involved as subjects in research but do not commonly participate as research partners in the development of interventions [9]. The competence and ability of children to participate in research is generally undervalued [10] and their involvement in research is therefore mainly passive [9,11]. This is even more apparent when research concerns children in sensitive contexts such as children survivors of cancer. Involvement of children in research in sensitive contexts is, however, important to ensure that developed resources meet needs based on the cognitive and emotional developmental stages of the children and their requirements on usability and experiential quality [12-14]. Children’s involvement in research is nonetheless disputed and treated with reluctance by some, especially when researchers base their resistance on the notion that involving children might adversely affect the accuracy or quality of the research or on ethical principles that participation might harm the child [8,14-16]. Most research with the purpose of developing health promotion interventions for children in health care is consequently primarily based on the involvement of parents, caregivers, and other stakeholders. However, adults’ views and experiences cannot replace the qualities that come with genuine involvement and partnership with children regarding their perspectives on health promotion and development of resources based on their own perspectives [9,11,17]. Thus, we still have some way to go before children’s participation in research is seen as a precondition for and a hallmark of quality and validity of research.

The objectives in this paper are twofold. First, we discuss the reasons for involving children in health promotive research and explore models for children’s participation in research. Second, we present how we involved children in the development of a digital peer support service and describe how different methodologies tackled challenges with involving children in a vulnerable context and how the methodologies corresponded to different levels of participation.

### Why Is It Essential to Involve Vulnerable Children in Health Promotive Research?

Increasing focus on the significance of involving children in research concerning themselves has contributed to less research being performed *on* children or from the parents’ or caregivers’ perspectives. Instead, studies with a child focus are increasingly being performed *with* and *for* children, with the children seen as social actors who are treated as experts on their own lives [17]. How children are viewed is essential to the discussion about children’s involvement in research. Children as social actors was emphasized by the Convention on the Rights of Children [18] in 1989 in what it described as the right of children to participate in and influence all matters that relate to them. This right includes both research and development that directly or indirectly affect children. Central principles state that all children have equal dignity and rights (Article 2), that children’s best interests shall be a primary consideration in all actions concerning them (Article 3), and that children have the right to shape and express their opinions and have them taken into account in all matters affecting them (Article 12). These principles are relevant for how researchers relate to children’s participation and serve as a standard for how integration and assessment of children’s participation in research should be planned and estimated.

Children’s participation in research is increasingly seen as essential for providing new knowledge and developing health promotion interventions that are credible from a child perspective [10]. This has led to the development of new methodological approaches that involve children throughout the research process instead of limiting their involvement to distinct parts of the research and to single methodological approaches such as observations, interviews, and questionnaires [19]. For example, an emerging field of participatory design methodology with children has been used to bring researchers and children together in a systematic process of collaboration for the design of health promoting interventions [12,13,20]. The participation of children in research can be achieved through involvement in different stages of the research process and for different purposes such as defining the need for research in a particular area, formulating research questions, planning and designing methodology, assembling and analyzing data, designing the proposed interventions, and giving recommendations for dissemination of findings [15]. However, the degree of children’s participation in stages of the research process depends on adults’ perceptions of their capabilities to participate and the importance of their participation for the quality and credibility of the research [8,15,16]. It is also affected by the trust between the researcher and the child [21] and the capability of the researcher to minimize the social differences between them. The researcher needs to reflect on research values, purpose, and methodological traditions [9] and be aware that children are representatives of a younger generation that in many aspects have other experiences, values, and goals than that of the adult researcher [17]. The researcher also needs to reflect that children’s involvement appears to evolve and progress over time [22]. It is essential that the level of children’s participation in research depends on the attitudes of adults around them [15] but also on the conditions and experiences associated with each child and her/his parents [8,23]. The notion of risk and the trust in the researcher and the institutions represented are vital for parents to give their permission for their children’s involvement in health research [21]. Perceptions among researchers that child participation can have a negative influence on the quality and credibility of the research are associated with participation that is limited to involvement without any real influence or relevance. Many researchers are dedicated to participative methods giving children opportunities to express views and share experiences. It is less common, however, that children are given the opportunity to have a significant role in the research design, data gathering, analysis, interpretation, and implementation of the outcomes of the research [9]. This article suggests that children need to be involved in research in its true sense in order for the researcher to be able to understand the context of the research area.

### Children’s Participation in Research: What Is It About?

#### Overview

Models that describe levels of participation are valuable as benchmarks in the planning of research and as guidance for evaluation of participation. There are a number of models available, some of which have been disseminated and are widely practiced (Table 1). Based on similarities and overlaps between these models, we have categorized different levels of children’s involvement into nonparticipation, consultative participation, and collaborative participation. These 3 categories represent condensations that we have made based on the different levels of participation that are described in these models. Nonparticipation can be described as situations where adults avoid involving children or involve them in ways that have no real impact on the research and even in ways that give a false semblance of partnership and real influence over the implementation of the research and the findings resulting from the research. Consultative participation means that adults seek children’s views in order to build knowledge and understanding of their lives and experiences. This is primarily done by inviting children to express their views and to provide data to the researcher. The children’s opinions are treated seriously, and the researcher gains knowledge and an understanding about their lives through this process. However, at this level of participation, children are not equal to the adult researchers and have no impact or control over the focus of the research and the outcomes and interpretations. At the collaborative level there is a greater degree of partnership between adults and children, where children have the opportunity to be actively involved and influence all stages of the research process. The partnership between the child and the researcher includes consultation, mutual trust, and shared decisions. Not only are children’s views taken into account at this level, the children are also involved in making decisions.

**Table 1:** Descriptions of levels of participation in various models of child participation in research.

Models	Nonparticipation	Consultative participation		Collaborative participation		
				Adult-initiated	Child-initiated	Jointly initiated
Hart [24]	Manipulation, decoration and tokenism	Assigned but informed	Consulted and informed	Shared decision with children	Directed by children (adults facilitate); shared decision with adults	
Treseder [25]		Assigned but informed	Consulted and informed	Shared decision with children	Directed by children (adults available); shared decision with adults	
Shier [26]		Listened to and encouraged to share views	Children’s views are taken into account	Involved in decision making; given power and responsibility		
Chawla [27]		Prescribed participation; assigned participation	Invited participation	Negotiated participation	Self-initiated negotiated participation; graduated participation	Collaborative participation
Reddy and Ratna [28]	Active resistance, hindrance, manipulation, decoration and tokenism, tolerance, indulgence	Assigned but informed	Consulted and informed	Shared decision with children	Shared decision with adults; directed by children (adults invited)	Jointly directed
Lansdown [29]			Children consulted and invited	Children collaborate with adults	Led or managed by children (adults support)	

The collaborative level includes different approaches regarding who initiates and directs the research process—adult-initiated, child-initiated, or jointly initiated. Even though the children have a decisive role in the collaborative participation, their participation is dependent on adults who support and facilitate their involvement. The goal of the collaborative participation level is to empower children to influence both the process and outcomes for any given research activity. According to the definitions outlined in the Convention on the Rights of Children [18], the minimum level for children’s participation in research dealing with themselves is collaborative participation [26]. This means that researchers should always strive toward integrating collaborative levels of participation in their research process where appropriate. For example, approaches that are normally used for consultative participation, such as interviews and questionnaires, can achieve the collaborative level of participation by giving children the opportunity to influence the content of the questions asked and the conditions under which the data collection is done. This influence gives them control over what the research is going to be about and how the research is going to be carried out.

The most widely referred model for child participation in research is that described by Hart [24], who uses a ladder to present different levels of participation. Hart’s model, as well as all the other models we have included, comprises 2 levels where children are informed at a consultative level and a third level where the child and adult collaborate through an adult-initiated process. Few models [24,25,27-29] include child-initiated collaboration, and only 2 models [27,28] describe jointly initiated collaboration in which adults and children work together to reach common goals. In the partnership of jointly initiated collaboration, both children and adults are empowered to play different roles and both share ownership of the process.

Some of the models are linear (starting with nonparticipation and continuing hierarchically with levels of gradually increased involvement) and imply that there is a goal to reach the highest level of participation [24]. Other models are circular (nonhierarchical) [25,27,29] indicating that each level has the potential to be the most appropriate for a given circumstance and therefore do not include a progressive hierarchy between the levels. The model by Chawla [27] urges that it is important to understand which degree of participation already occurs in formal and informal settings as well as taking the children’s existing life experiences into account. The model by Lansdown [29] emphasizes that the child’s participation should be introduced as early as possible in the process and with as much control as possible. The models by Chawla [27] and Treseder [25] also highlight that children can go from 1 level of participation to another when they progress in competence and ability to participate. All models describe assessment of when and how to involve children in the process, but 2 models [26,29] have a stronger focus on practical planning and evaluation of situations where adults work with children. All the models are based on the identification of the appropriateness of different levels of participation based on the conditions and experiences of the children involved and that forms and levels of participation should be adapted to the various activities taking place in the research process. The models should thus not be used as tools to focus participation toward the highest level of involvement but as supports for researchers on involving children in each phase of the research process at a level that is possible and appropriate for the best interest of the child. However, it is important to be aware of how the power relationship between the researcher and the participating child could be balanced so that children have a genuine possibility of influencing the research process. If not, there is a risk of tokenism where the children are given a voice and opinion but have little if any real influence on discussions and decision making. The authors of the models state that the outcome of children’s participation in research is active citizenship and democracy.

As outlined above, these models for children’s participation in research describe different levels of participation from nonparticipation to consultative and finally collaborative participation. But none of the models alone describes the content in all these levels, and there are ambiguities as to how the different levels relate to the requirements on participation that are declared in the Convention on the Rights of Children and on what grounds a certain level of participation is desirable in a certain situation. This shortcoming complicates the application of the principles of child participation in planning, implementation, and dissemination of research and knowledge that relate to children and that are based on their participation. We therefore propose another more practically concrete model that is inspired by the work of the International Association for Public Participation and includes both the consultative (inform and consult) and collaborative (involve, collaborate, and empower) levels of participation. Below we describe the 5 levels of participation in this model and why these can be used to reach certain goals for the research, what promises that are made to the children involved, and how the children are practically included in the research process (Table 2). An important point with this model is that although the highest levels of participation are found among the collaborative types of participation, it also shows how and why to optimize children's participation to the most appropriate and feasible level of participation. This can help researchers in increasing children’s participation in areas of research where they have primarily been involved as informants and consultants.

**Table 2:** Levels of participation in the research process.

	Consultative participation		Collaborative participation		
	Inform	Consult	Involve	Collaborate	Empower
The goal with child participation	Respecting children while keeping them informed	Preparing and performing research based on children’s views	Involving children throughout the research process	Making decisions with children throughout the research process	Enabling children to be involved in making final decisions throughout the research process
The promise to the child	You have access to all information and have been informed equally as much as anybody else in the project.	You will be important for information-seeking and feedback throughout the research process.	You will work together with us to help and contribute with your perspective on the research.	You will be an equal partner in finding and developing solutions in line with the purpose and aims of the research.	Your efforts and contributions will be visible and implemented in the outcomes of research.
Activities to involve the child	Explain to the child what is to be done, how and why, as well as the consequences of participation.	Study the child’s experiences through interviews, observations, and questionnaires.	Work with the child in workshops and other activities in which they are allowed to contribute with their perspectives.	Involve the child in workshops and other activities in which they are allowed to ideate, create, contribute, test, and evaluate.	Present to the child which contributions they have made to allow them to elaborate and confirm.

#### Consultative Participation: Inform

Children have fundamental rights to be informed of everything that involves them—including research [18]. In participatory research, the goal should be to provide the children with information on a level commensurable with their age and cognitive skills and having in mind their potential vulnerability as children and end-users [12,13]. This means that efforts have to be made to adapt information both regarding the format of the information and the appropriateness of its content. It is equally important to make it clear for the child that he or she has access to all information and has received as much information as everybody else who is involved in the research: other children, their parents, stakeholders, or others [29]. The children also have the right to know what their involvement will be and the consequences of participation.

#### Consultative Participation: Consult

An important aspect and value with child participation is their contribution to the identification of the purpose and aims of research and in obtaining feedback on plans, data collection, and interpretations [30]. There are sometimes concerns that the increased involvement of children in research can compromise the quality and credibility of the data. However, our stance is that the involvement of the target group in the formulation of purpose and research questions is essential for the validity of the research approach. It not only provides justification that the research is relevant and important but also contributes important input to formulating the purpose of the research, which methodological choices should be made, which participants are most appropriate to recruit, and when and how they should be involved. The consultation of children from the start of the research process thus sends a signal that children have an important role in defining what the research should be about and how it should be performed. This is valuable for the researcher-child relationship, the continued communication with stakeholders during the research process and when disseminating the results to the participating children, and the intended target group and stakeholders who are involved in the research process or are the beneficiaries of the research outcomes.

#### Collaborative Participation: Involve

Using tangible ways to ensure that children’s goals, concerns, attitudes, and preferences are understood and reflected in the outcomes of a project also promotes the participating children’s development of skills and confidence in how to contribute toward the purpose and aims of research. The aims of such activities are for the children to work together with the researchers to help and contribute with their perspectives on the research. This could be done by using a playful and creative approach during interviews and workshops, for example, through storytelling, photography, and drawing activities. In order to ensure that the children’s involvement will be valuable and significant for the outcomes and progression of research, it is important that the planning of when, where, and how children are involved is carefully considered in relation to their age and abilities, not least to ensure that their participation is not of a decorative or manipulative nature and to avoid their rights or integrity being violated through their participation [24,29].

#### Collaborative Participation: Collaborate

To ensure that child involvement does not finish with being consultative and limited to informing or merely supporting researchers in the research process, the researchers need to find ways to involve children in decision making throughout the research process. At this level, the adult researchers share ownership of the research with the children and ensure that the children have real opportunities to influence the research process and outcome [31]. This requires a different mind-set from the adult researcher and requires that the children be invited to play a significant role in the codesign of research [9]. Having children involved in actual decision making indicates that they are viewed as important and equal partners in finding and developing solutions in line with the purpose and aims of the research [24,26].

#### Collaborative Participation: Empower

Empowering children through participation means allowing children to take an active role and have influence when making final decisions in both process and outcomes in any given research activity [26]. In order to achieve this, adults interacting with the children have to be credible and trustworthy so that the children’s efforts and contributions become visible and implemented in the outcomes of the research. At this level, an increase of self-directed actions and final decision making lies predominantly in the hands of the children. The overall goal with empowered children is that they can become active advocates for the realization of their own rights and can play a useful citizenship role in their community. Final decision making does not necessarily mean that children make decisions that adults commit to follow. Taking part in final decision making can also mean helping adults to make decisions by participating in analysis and interpretation or to be given the opportunity to provide feedback on or confirm decision made by adults.

## Methods

### Case Description and How We Involved Children Cancer Survivors in Research

We applied our model for children's participation in research to our development of a digital peer support service for children cancer survivors. The model helped us in guiding selection of the most appropriate methodologies for each step of the design and research process to ensure both feasibility of the process and involvement of the target group. The design and research process resulted in a digital peer support service adapted to the needs and preferences of the target group. The service was in the form of a mobile app called *Give Me a Break.* The app provides an interactive platform for play and social interaction that is a safe meeting place where cancer survivors (8-12 years) can interact with peers, find new friends, and build long-lasting friendships. The platform is composed of a virtual playground that connects users and provides creative playful activities facilitated by an online youth worker with the objective of stimulating interaction and integration of social media applications and thereby encouraging continued interaction using other social media channels or other venues. The service is introduced at discharge from intensive care or during clinical check-ups at the hospital following completion of treatment. In this section, we describe how we worked to involve the children in this process and how their involvement made it possible for us to focus on the children’s perspectives in the design of a digital service that relates to their goals, attitudes, problems, and frustrations and that meets their worldview, their cognitive and emotional developmental stage, age, and gender. More detailed descriptions of the methodology used and evaluation of the validity of the methodology for supporting different levels of children’s participation in the research have been presented elsewhere [5,20,32-35].

## Results

### Inform: Respecting Children While Keeping Them Informed

Before we could inform and ask the children about their willingness to participate in our research, we asked the adults around the children. A key factor for having children participate and interact with adult researchers is that gatekeepers give them the possibility and permission to do so. Most important for this is that parents give permission for their children’s involvement, and this is strongly dependent on trust they have in the researcher and the associated institutions [21]. Research that addresses child health and how this can be promoted can be legally, ethically, and morally complex and therefore requires a dialogue with stakeholders and gatekeepers in order to establish a suitable level of involvement of the children. In our case, we first had discussions with representatives from the pediatric health care services, and they confirmed the need for the proposed research and the planned approach. These health care professionals surveyed parents and children concerning whether they wanted to participate. The parents’ positive attitude to allow their children to participate in our study was based on trust in our roles as scientists and trust in health care professionals in terms of their approval of our research.

We made efforts to provide information in formats that were appreciated by the children. During project initiation, this meant that information was available to the participating child in age-appropriate fact sheets, illustrations, and websites. An important aspect of the information during project initiation was that the information letter and consent form were designed and formulated in a way that made it clear to the child the aims and activities they agreed to participate in. The children and parents were first given an invitation to participate through their nurse and then through age-appropriate written information and consent forms. This increased the likelihood that the information was appreciated and that the message was understood. It also showed the children that we were interested and committed to reaching the child in a way that was appropriate for them. The children were also given the opportunity to sign the consent form even if it was not needed in a legal sense. This was important to convey our ambition that the children were to be active participants in the research and that they had the right to decide on their participation. This way of recruiting participants to the study resulted in that several eligible participants opted out of participating in the study either indirectly as the result of judgments made by representatives from the pediatric health care services or directly based on considerations or decisions made by the parents or by the children themselves.

The researcher responsible for collecting data in the form of questionnaires, interviews, or workshops carefully planned (through review of the literature and several workshops with the research group) how to provide information in a child-friendly way and which type of data was to be collected and why and how. For example, we have seen the importance of using schedules that can be placed on the table in front of the children or posted on the wall of the premises in which the activities take place so the children can keep track of what they are doing and where they are in the time plan. The children appreciated these schedules because it made clear what should be done during the meeting and invited the child to resume with previous questions or ask new questions as work progressed.

Another example relates to data collection; it can be important for the child to feel in control of what information is documented and how. This affects, for example, how scientists can document with field notes without the child feeling studied and manipulated by the adult. It also affects how to suitably use the recording of sound and images and explain how this type of data is stored and who has access to the recorded material. These are important details for children, who are increasingly aware of the risks in relation to how information about them is documented and shared among others. To ensure that the children in our study felt that they had control over the documentation, we started the data collection with an explanation about underlying principles of their participation and how we planned to document the process and that only members of the research team had access to the data. All our methods used at this level of participation are summarized in Table 3.

**Table 3:** Consultative participation: inform.

	Invitation			Data collection		
The methods used at this level	We gave information to and collected permission from parents and representatives from pediatric health care.	Children and their parents were given verbal invitation to participate by their nurse.	We designed and formulated information letter and consent form in a child-friendly way.	We gave verbal explanation of what data will be collected, why, and how.	We gave verbal explanation about underlying principles of their participation and how we planned to document the process.	Schedules of activities were used so that the child could keep track of what is to be done.

### Consult: Preparing and Performing Research Based on Children’s Views

From a participatory design approach, the users should also have impact on the purpose and design of the project [13]. Our research project was prepared and initiated based on needs of peer support formulated from national cohort data of young adult cancer survivors [33], as well as a blog observation made on the Swedish Childhood Cancer Foundation website. Based on the observed blog post, we documented how an adolescent girl described that she really wanted a cancer friend and a peer to talk to. We then used pilot studies with individual interviews with young adult cancer survivors, parents, and clinicians in order to gain insight into the problem and get an understanding of which research question should be used to provide more information and create an understanding of the phenomenon. By presenting these research-driven objectives and ambitions in a very preliminary form to the potential user group, we were able to redefine our initial plans based on key input from the potential users. This ensured that we from the beginning framed the research in line with the needs and preferences of the target group.

For data collection there is a need to establish a strategy for how children's perspectives are to be taken into account. This can be done together with representatives of the target group or be influenced by other children from the same age group. The main thing is that researchers choose a data collection strategy that gives children the best opportunities to participate with their opinions and share information while safeguarding their interests and rights. The difference in the power relationship that exists between children and adults cannot be overestimated [36]. Since the experience of this power imbalance differs between individuals, research should be designed to safeguard that children are treated with respect and to prevent them from feeling subordinated or exploited. Such feelings, even if they are caused unintentionally, hamper the children’s ability and motivation to participate in the research. In planning our research interviews, we have been careful to choose premises familiar to the children in order to give them a safe environment to work in. We have also been careful with how we dress, the language we use, and how we behave in order to avoid using or strengthening markers of power that the children recognize from school, health care, or society. We planned the timeframe for data collection and for working together in workshops adapted to the children's life world, such as school hours and holidays and hours of the day that best suited the children. This meant that the planning was done based on when the children had the opportunity to participate and when they had optimal capability to mobilize the most energy and commitment for their participation. All these considerations were important to ensure that the children could participate in the research as equal partners and also felt that they were seen as equal partners by the adult researchers. At the beginning of each interview and workshop sessions a range of icebreaking activities, such as exercises to get to know each other by talking about things we like to do or movies or music we like, were used in order to build trust and a relationship between adults and children. We were also careful to find a balance between how many adults and children were involved simultaneously and made sure the adults involved did not vary over time so it was possible for children and researchers to establish a rapport and relationship.

As outlined above, the information given to the child during consultation is crucial for the child’s ability to be involved. Similarly, the methodological setup for how the consultation is carried out should be adapted to the needs and existing life experiences of the participating children. When we organized focus groups and design workshops we evaluated our design through piloting to assess the feasibility and relevance of the outlined activities and content related to the preferences and experiences of the target group.

In order to gain an understanding of children's views of the defined phenomenon, we started with focus groups and interviews divided into 2 separate sessions with children between the ages of 8 and 12 years with experiences of cancer treatment. The interviews focused on the children’s own experiences of friendship and peer support in the context of everyday life following cancer treatment. The focus groups had a semistructured approach and were divided into 3 phases: (1) an icebreaking phase with “get to know” exercises, (2) a discussion phase centered on friendship, and (3) a closure phase where the session was summarized and where both the children and researchers had the opportunity to reflect on their participation. At the end of the first session, the children had the opportunity to suggest discussion themes for the upcoming session. Data analysis was done by the researcher and was based on children’s views during the interviews. The methods used at this level of participation are summarized in Table 4.

**Table 4:** Consultative participation: consult.

	Establishing the idea, purpose, and design		Data collection		
The methods used at this level	We based our purpose on needs formulated from national cohort data of young adult cancer survivors and from empirical data collected from blog observations of users.	Individual pilot interviews were done with young adult cancer survivors, parents, and clinicians.	We designed approaches to avoid power imbalance and to support motivation to participate.	Pilot interviews were done with children to validate the interview techniques.	Semistructured focus group interviews were done with the purpose of getting children’s views on the phenomenon.

### Involve: Involving Children Throughout the Research Process

In our project, we have seen that a combination of involvement of different parties may be the best solution to achieve as high a degree of participation as possible. There can, for instance, be occasions when children are not expected to understand the problem or context as a whole, such as a complicated treatment process, but where the researcher still wants to take measure of their perspectives, perceptions, and experiences. In such contexts, we designed plans and methods for how to involve the children as much as possible, for example, by interviewing children along with their parents to supplement the children’s experiences with their parents’ experiences in those respects which the children are not able or willing to participate. Another solution that we used was to strategically select areas of research in which children legally, ethically, or morally are not considered to be mature enough or able to participate and in these cases use stakeholders as proxy informants to complement these areas in which children cannot participate. An example of this could be to use stakeholders as informants about possible causes of ill health in the target group and to use children from the target group as informants around what can promote their health. For both of these strategies, the interpretation of the knowledge obtained must be carefully balanced so that the results reflect a reasonably accurate picture of the children’s reality. Thus, the researcher has to ensure that the stakeholders’ contributions are not excessive or contribute things that are not supported or appreciated by the children, identify any misinterpretations or errors, and improve or add details that have been missed.

A further aspect of what promotes a consultative participation of children in research is how we as adults create conditions for meeting the children on a level that is appropriate based on their daily lives and their abilities and interests. The importance of choosing a playful and creative approach during interviews and workshops cannot be overstated [37]. For example, we used photography and drawing as a complement to common interview questions for data collection in order to encourage the children to express their views and experiences. We also used design-oriented iterative workshops to include children from the target group in the analysis and processing of quantitative or qualitative information obtained. These workshops helped to include the children’s perspectives when interpreting the data and base further development on contributions from the children themselves. In order for this to work, we used methods to support children’s participation (eg, brainstorming and sketching) where children came with ideas and suggestions for solutions that the researchers together with designers elaborated on and embodied in sketches or low-fidelity prototypes that were then iteratively refined together with the children. In the first workshop each child-adult pair created a character that was presented to the rest of the group. This work visualized basic demographic information, personal values, and motivational aspects of the user group. After the workshop, the characters were compiled into proxy personas by the researchers and these were used for creating storyboards. Each storyboard illustrated a redemption scenario based on the characters the children created during the first workshop and that was used as working material in the subsequent workshops to obtain the children’s perspectives on solutions to challenges and problems of the proxy personas highlighted in the scenario descriptions. This approach made the children’s contribution to the research and design process concrete and tangible and helped us in making the children’s participation visible in the final material. This iterative (4 workshops) collaboration with the target group also made it possible for us to continually get feedback on the focus of the research and the results and ensure that the design process was appropriate. Children could, through their role as both informants and consultants, help us with deciding to continue on a path or if alternative directions or strategies needed to be taken.

To evaluate the prototype we developed based on the compiled empirical data, we involved children in usability tests, a user diary study, and a follow-up focus group interview. During the usability test sessions, children were individually given tasks to perform to evaluate functionality. A facilitator guided them throughout the tasks. The second part of the evaluations consisted of a 2-week use study where the children who tested the prototype were given diaries with questionnaires to fill out each day. After the use study was complete, children were invited to participate in a focus group interview around their experiences of the use of the prototype. All the methods we used at this level of participation are summarized in Table 5.

**Table 5:** Collaborative participation: involve.

	Optimizing children’s participation		Evaluation of the prototype		
The methods used at this level	We combined participation of children and proxy informants during the research process.	We created playful and creative material and approach during data collection.	We used usability tests.	We used a 2-week use study with diary documentation.	We used a follow-up focus group interview.

### Collaborate: Making Decisions With Children Throughout the Research Process

We have used a collaborative approach for data collection to involve children not only as informants but also as important and equal partners in decision making and design. Through the use of iterative design workshops, we were able to collaborate with children, starting with very general contribution to defining the user group, continuing with defining goals, problems, and frustrations of this group and, finally, identifying and designing solutions for how to support or help the user group in the best way. By making this into an iterative process, we as researchers were able to summarize and prepare outputs from each workshop until the next session. Cooperation with the children from the target group in several iterative steps allowed for the children to initially take a fairly simple role that did not entail a high degree of independence but rather a dependence on the support from a collaboration with other participants, both adults and children. As the children became experienced and confident in the role as co-creators and increasingly familiar with the complexity of the challenges of the research topic, they developed an increasingly independent role and were able to take more responsibility to contribute to the process going forward toward the purpose of the research. During this development of the child’s ability and independence as co-creator, it is important that the support from and collaboration with adults is adapted and changed as the work moves forward. One should not underestimate the importance of support from adults to initially help the child with understanding the meaning of the activities and with assisting the child in the informative or creative activities. The support from adults must thus initially be quite extensive and thereafter gradually reduced as the child develops confidence and experience [20]. One should, for example, not be afraid of initially pairing each child with an adult, as they might not be able to participate without such support. Such a high degree of adult involvement and support can thereafter be reduced to finally be at a minimum level. In order to support this, we used a co-creation process where the first session dealt with defining and describing the target group and the aim of the research and where outcomes were the result of the work from pairs of a child and an adult. The outcomes of several such pairs were then summarized and formed the basis for the next session where the children could take a more independent role.

In projects where the children have a high degree of participation during data collection, it is common that this participation is interrupted when the data analysis phase begins. In our project we have, during analysis and implementation, used summaries and abstractions of qualitative data, compilations of statistical data, or sketches and prototypes and invited the children who participated in the data collection to comment and provide feedback on these outputs. This interaction has been designed in the form of joint workshops, individual follow-up interviews, or written or digital demonstration of summations, models, sketches, or prototypes. Activities have been arranged either as single events or as repeated short interactions with children. The main purpose of this iteration is to offer children the opportunity to provide feedback on the conclusions and implications that have been made based on the data they participated in gathering, contribute with essential information for the continuation of the research and design process, and finally assess the research and design outputs through prototype usability evaluation. The opportunity to provide feedback and to continually evaluate the outcomes in the research process ensures that the results of the research are credible and based on the objectives that the children have with their participation. All the methods we used at this level of participation are summarized in Table 6.

**Table 6:** Collaborative participation: collaborate.

	Iterative design workshops			Follow-up and feedback workshops with children
The methods used at this level	We performed workshops to build familiarity and create proxy personas.	We used workshops to co-create redemption scenarios.	We used workshops for feedback and prototyping.	We used workshops to verify and further develop ideas. The children worked in teams and moved between stations with low-fidelity prototypes on which they gave verbal and drawn feedback.

### Empower: Enabling Children to Make Final Decisions Throughout the Research Process

In order to connect the empirical findings from the previous steps into a coherent model that could effectively and efficiently drive the design of a health promoting service for the children in the target group, we used summaries of key traits of the user group to construct personas to capture the human-centered values in the project. The persona is a model of a user archetype that is based on empirical data and focuses on behaviors and goals of the users in the target group [38]. The activity of generating personas is both analytical and creative. The children were involved in this work by the shaping of characters describing demographic information, values, and motivational aspects that were used as a foundation for proxy-personas used in redemption scenarios [20]. The final personas were created based on the documentation of children’s reflections and discussions of the scenarios. Supported by our personas and accompanying context and key path scenarios describing the use of the digital service that we wanted to develop, we were able to keep the interests of the user group in focus during prototyping by aligning all design ideas with the goals, preferences, attitudes, and frustrations described for our personas. In doing so we sought to ensure that the interests of the user group were integrated into the scenarios that were used during the prototyping and implementation phases of the project. The use of personas, co-created with the children, indirectly involved the children in decision making during the design process and prevented ideas, conclusions, or initiatives that were based on the empirical data and appreciated by the researchers and designers but were not in line with the developed personas.

At the end of both the design and prototyping phases of the project, outcomes such as descriptions, visualizations, sketches, mock-ups, and prototypes were presented to be verified by the involved children for feedback and confirmation or identification of further design directions. For example, following the focus group sessions and design workshops, all summarized data, the completed personas, and the first design directions of the prototype were presented to the participants and their parents at a workshop. The feedback and responses at this session were documented and used for continuing the research and design process. Similarly, at the end of the prototype development, a printed outline of the purpose and functionality of the service was presented to the participants, and the aesthetics of the design were presented on a website with illustrations, screenshots, and movie clips. All participants were given the opportunity to evaluate the prototype design and relevance in accordance with the purpose of the project and their individual experiences and preferences. These final iterations were important not only to ascertain quality and relevance of outcomes but also to consolidate the participant’s role as partners in the research and design and to show the importance of their contribution for the outcomes of the project. All our methods used in this phase are summarized in Table 7.

**Table 7:** Collaborative participation: empower.

	Personas	Validation workshop with children and parents	
The methods used at this level	We co-created personas that throughout the process kept the interests of the children in the focus of the researchers.	We performed a workshop to verify that the outcomes of the research were in line with the goals and concerns of the target group. Children and their parents gave verbal feedback on confirmation or identification of further design directions for the ideas and prototypes presented at each station.	The participants involved in the initial focus groups evaluated the outcomes of the research in the form of a printed outline of the purpose and functionality of the service; a website presenting the aesthetics of the design with illustrations, screenshots, and movie clips; and a form evaluating the prototype design and relevance to the purpose of the project and individual experiences and preferences.

## Discussion

### Lessons Learned

The benefit of our research was the combination of a variety of methods to reach the most appropriate level of participation for the children in relation to the purpose of each part of the project. We believe that the combined approach in our study demonstrated that children’s participation in research is an important quality indicator for the research process and for the outcome of the research. Our intention with this paper was not to evaluate the validity of our model as such but rather to practically show how we implemented the model and our experiences of how this supported us in incorporating the children’s perspectives to our research process. The methodologies described in this paper have been evaluated in several studies describing the different phases of the research project. These studies have evaluated the feasibility of involving the children in the research process as well as the importance of their involvement for the research outcomes [5,20,32-34]. Evaluation of implementation of this model in projects with other objectives in other contexts is needed to keep it up to date and to further prove its validity. Some experiences from our work on the project are worth mentioning.

One practical challenge when including children in research is that gatekeepers have a role in limiting researcher access to participants [8]. In our case, we believed it was crucial for children’s participation that we convincingly could describe the forms of participation to parents and stakeholders in the health care system and support them and the children themselves to participate. We achieved this primarily by being clear in the information we shared with parents, stakeholders, and children by adapting this information to each target audience. We also put effort into involving health care professionals, who had responsibility for the children’s care process, early in the planning of the research in order to establish trust with the health care professionals and the children’s parents.

Children’s right to refuse participation is another area of significant challenge. There could be riskiness to health care professionals or parents using their influence to convince the child to participate in a project if they support the idea of the project [9]. There could also be a risk that the children choose to participate only because they want to please their parents. To counteract this, we made efforts to give every child age-appropriate written and verbal information and give them the opportunity to sign the formal informed consent in order to display that they themselves have the right to decide if they wanted to participate or not. In some cases, the children wanted to participate but not the parents, and in some cases, the parents wanted to participate but not the children. In both these cases, it ended up that these children were not included in the study. The children who chose to participate expressed that their motivation to participate was that it could be fun and they wanted to contribute to the improvement for other children who share their experiences.

One major challenge when working together with children in research is the power imbalance that exists between the adult researchers and the children. In our case, we made up a strategy for ensuring that we treated the children as equal partners in the different stages in the project. To work toward achieving this, we had several discussions in the research group at the beginning and throughout the project in order to be aware of our own assumptions and behaviors. We made efforts, in the way we were dressed, the language we used, and the way we behaved in the group of adults and children, to minimize any experience of power imbalance between us. We also put efforts into allowing all parts of the project to take the time that was needed for the children to feel they had the opportunity to contribute and that there would be enough room for the children to iteratively verify the outcomes of the decisions taken together with them. This meant that the project went on for a longer time period than first anticipated (3 years instead of 2) and involved more interactions than if the children’s participation would have been only consultative. The extensive time spent on interaction between the children and the researchers and designers posed a challenge to the project in several ways. For example, we as researchers had to adapt our traditional research process to a work process that took into account that all parts of the project would be iterated with the children and timed with their life world. Research funders and stakeholders had to be continuously informed of the progress of our work and the value of using substantial time for interaction with the children in the research. Finally, the children’s interest in participating depended on their feeling that it had a significant impact on the outcomes. A further difficulty with running a project over a long period of time (3 years) was that the oldest children eventually aged out of the intended target group for the project and their interest in working with the younger children changed as they reached adolescence.

### Implications

The model described in this paper and the experiences from our case can inform researchers in their planning of strategies for children’s participation in research. Increasing the level of children’s participation is a valuable asset in the development of digital health promotion interventions for children since it brings in user perspectives that are essential for the design of relevant and functioning services that meet user needs and are adapted to user preferences. Furthermore, being able to implement a model-based structure for the why, what, and how of children’s participation facilitates connecting with stakeholders and gatekeepers for child participation in the initiation of a project.

In our project we have involved children in all phases of the research process in order to understand their motivations, behaviors, and preferences and to ensure the outcome of the research is in line with the goals and needs of the children. There are some significant challenges in involving children in the research process that are valuable to pay attention to [10,24]. The challenges include how to achieve an appropriate level of participation during a research project and how to create opportunities for children to feel that they understand and have genuine possibilities to influence the research process [8,14]. One important issue for children’s participation in research is to assure that they understand what they are involved in. It is important to not only provide information to their parents but to also inform the children in an age-appropriate format and in a way that makes it clear to the child what purposes and activities they agreed to participate in. Similarly, even if formal informed consent from the children is not needed in a legal sense for them to be involved in research, this requirement signals an intention from the researchers that the children are active participants in the research and that they have the right to decide on their participation. Another issue that needs consideration is that the goal with involving children in research is not necessarily to reach the highest but rather the most appropriate level of participation. The linear composition of many of the models described in Table 1 does not capture the complexity of children’s abilities and prerequisites for participation. The opportunities for children to achieve the empowerment level (described in Table 2) during the research process is a matter of time and trust. Through a process of iterative meetings with the researchers during a prolonged time period where the children can see in what ways their contribution has meaning for the outcomes, they gradually progress in capacity to contribute toward the objectives of the research. This means children need possibilities for participation in research over time to build trust in the researchers and to develop abilities to make decisions along the research process.
